# Dapagliflozin effects on exercise, cardiac remodeling, biomarkers, and renal and pulmonary function in heart failure patients: not as good as expected?

**DOI:** 10.3389/fcvm.2025.1542870

**Published:** 2025-03-17

**Authors:** Massimo Mapelli, Irene Mattavelli, Elisabetta Salvioni, Nicolò Capra, Valentina Mantegazza, Anna Garlaschè, Jeness Campodonico, Filippo Maria Rubbo, Chiara Paganin, Teresa Maria Capovilla, Alessandro Alberto Nepitella, Rebecca Caputo, Paola Gugliandolo, Carlo Vignati, Beatrice Pezzuto, Fabiana De Martino, Giulia Grilli, Marco Scatigna, Alice Bonomi, Gianfranco Sinagra, Manuela Muratori, Piergiuseppe Agostoni

**Affiliations:** ^1^ Centro Cardiologico Monzino, IRCCS, Milan, Italy; ^2^ Department of Clinical Sciences and Community Health, Cardiovascular Section, University of Milan, Milan, Italy; ^3^ Cardiovascular Department, “Azienda Sanitaria Universitaria Giuliano-Isontina,” Trieste, Italy; ^4^ Cardiology Division, Nostra Signora di Bonaria Hospital, San Gavino Monreale, Italy; ^5^ Department of Cardiology, Casa di Cura Tortorella, Salerno, Italy

**Keywords:** dapagliflozin, SGLT2-i, heart failure, cardiopulmonary exercise testing (CPET), reverse remodeling, HFrEF

## Abstract

**Background:**

Sodium-glucose cotransporter-2 inhibitors (SGLT2-i) are standard therapy for heart failure (HF). We performed a holistic evaluation of dapagliflozin, including its effects on exercise performance, left ventricle (LV) reverse remodeling, cardiac biomarkers, fluid retention, and renal and pulmonary function.

**Methods:**

We enrolled HF reduced ejection fraction (LVEF) outpatients (EF <40%) eligible for SGLT2-i and performed cardiopulmonary exercise tests (CPET), pulmonary function tests, bioelectrical impedance vector analysis, and laboratory and echocardiographic assessments at baseline (*T* = 0), after 2–4 weeks (T1), and after 6 months of treatment (T2).

**Results:**

None of the patients interrupted SGLT2-i for adverse events albeit follow-up was completed by 67 of 75 enrolled patients. At T2, mean LVEF increased (from 34.6 ± 7.8 to 37.5 ± 9.2%; *p* < 0.001) while end-diastolic (EDV) and end-systolic (ESV) volumes decreased [EDV: 186 (145–232) vs. 177 (129–225) mL, ESV: 113 (87–163) vs. 110 (76–145) mL; *p* < 0.001]. Peak oxygen intake was unchanged [peakVO_2_: 16.2 (13.4–18.7) vs. 16.0 (13.3–18.9) mL/kg/min; *p* = 0.297], while exercise ventilatory efficiency (VE/VCO_2_ slope) improved [from 34.2 (31.1–39.2) to 33.7 (30.2–37.6); *p* = 0.006]. Mean hemoglobin increased (from 13.8 ± 1.5 to 14.6 ± 1.7 g/dL; *p* < 0.001), while renal function did not change after a transient worsening at T1. NT-proBNP, ST-2, and hs-TNI did not change as overall body fluids and quality of life assessed by KCCQ. NYHA class improved (*p*=0.002), paralleled by a decrease of MECKI (Metabolic Exercise test data combined with Cardiac and Kidney Indexes) score, from 3.3% (1.9–8.0) to 2.8% (1.2–5.7), suggestive of a positive impact on 2 years prognosis (*p* < 0.001).

**Conclusions:**

Dapagliflozin induced positive LV remodeling, improvement of exercise ventilatory efficiency, and NYHA class but without peakVO_2_ fluid status and cardiac biomarkers changes.

## 1 Introduction

Dapagliflozin is a molecule belonging to the class of sodium-glucose cotransporter-2 inhibitors (SGLT2-i). This type of drug, initially used in the treatment of diabetes mellitus, has demonstrated significant clinical and prognostic benefit over the past several years in patients with reduced ejection fraction heart failure (HFrEF) even in the absence of diabetes mellitus (1, 2). HF guidelines have taken up this evidence suggesting the use of SGLT2-i therapy in patients with HFrEF (3, 4). More recently, this drug class has also demonstrated significant prognostic improvement in patients with HF with preserved and mildly reduced systolic function (5, 6) and in chronic kidney disease (7). Considering the recent introduction of the drug into clinical practice, direct field evaluation is very important to refine clinical management of patients treated with SGLT2-i and to understand the mechanism behind the clinical benefits. Indeed, in some small preliminary studies performed in patients with HFrEF, SGLT2-i, together with an otherwise optimized medical therapy, have shown to be effective in improving left ventricle ejection fraction (LVEF) and other echocardiographic parameters of ventricular remodeling (8, 9). However, a holistic evaluation of the potential effects of SGLT2-i therapy on body function, including exercise capacity assessed by the gold standard cardiopulmonary exercise test (CPET), pulmonary function, body fluid homeostasis, and biomarkers have not yet been reported in patients with HFrEF.

The present study was designed to evaluate changes in CPET-derived parameters, pulmonary function, echocardiographic parameters of LV systolic function, cardiac biomarkers, fluid homeostasis, and quality of life (QoL) in a single-center cohort of patients with HFrEF (NYHA functional class II–III) treated with dapagliflozin.

## 2 Methods

At the HF Unit of the Centro Cardiologico Monzino, IRCCS in Milan, we enrolled a cohort of stable HFrEF outpatients who were eligible for treatment with SGLT2-i. The inclusion criteria were as follows: age >18 years; stable clinical condition defined as absence of heart failure exacerbations in the past 3 months, i.e., no hospitalizations for heart failure requiring intravenous diuretic administration; LVEF ≤40% (echocardiography); and diagnosis of HFrEF (3). In addition, they had to be able to undergo CPET and provide signed informed consent to participate in the study. The exclusion criteria included contraindications to SGLT2-i, moderate-to-severe chronic obstructive pulmonary disease (COPD), or an estimated glomerular filtration rate (eGFR) <30 mL/min/1.73 m^2^ according to Modified Diet in Renal Disease (MDRD) criteria (10). Patients who met the study’s inclusion and exclusion criteria underwent an initial evaluation (T0) that included:

• A clinical examination
• Kansas City Cardiomyopathy Questionnaire (KCCQ-12) to assess quality of life (QoL)
• Blood sample: complete blood count, creatinine, urea nitrogen, sodium, potassium, glycated hemoglobin (Hb), N-terminal BNP (NT-proBNP), suppression of tumorigenicity 2 (ST-2), high-sensitivity C-reactive protein (hsCRP), and high-sensitivity troponin I (hs-TNI)
• Standard spirometry
• Maximal ramp-protocol CPET on cycle ergometer
• Transthoracic echocardiogram
• Bioelectrical impedance vector analysis (BIVA)

**Figure 1:**
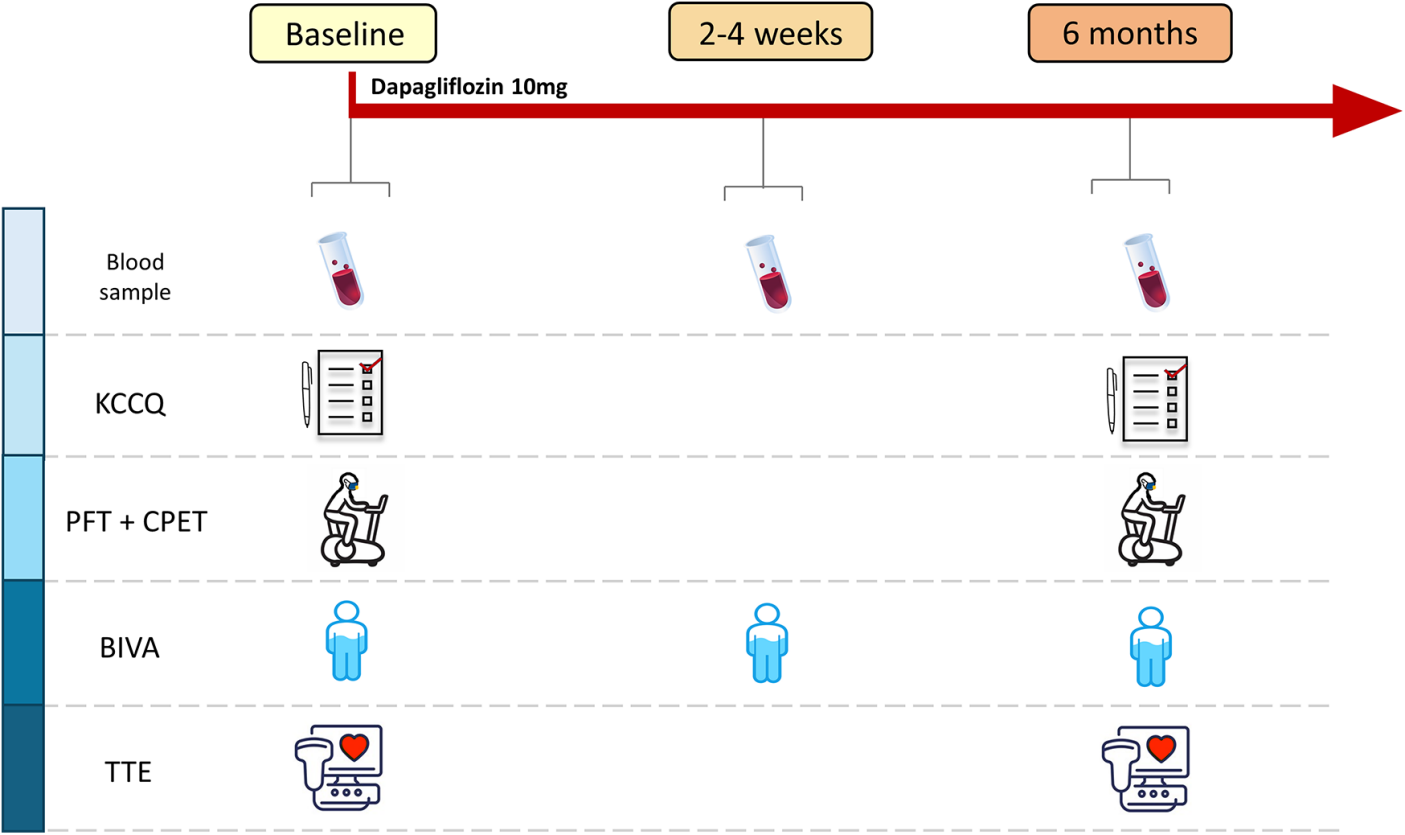
Study design. Scheduled activities at each study point. KCCQ, Kansas City Cardiomyopathy Questionnaire; PFT, pulmonary function test; CPET, cardiopulmonary exercise test; BIVA, bioelectrical impedance vector analysis; TTE, transthoracic echocardiography.

Subsequently, patients were prescribed dapagliflozin at a dose of 10 mg/day. Between 2 and 4 weeks after starting the therapy, a safety evaluation was performed, including a clinical evaluation and blood tests. All parameters evaluated at T0 were reassessed 6 months after the start of treatment (T2). The study synopsis is shown in Figure 1.

### 2.1 Kansas City Cardiomyopathy Questionnaire analysis

QoL was evaluated using the KCCQ-12 at baseline and at 6 months, administrated before any other assessment. The KCCQ-12 was analyzed combining the reported Physical Limitation, Symptom Frequency, QoL, and Social Limitation scales into the Summary Score, calculated as the average of the four scores. To calculate the summary score, at least one of the four scale scores must be present (11, 12). Only scales available at both T0 and T2 were considered.

### 2.2 Cardiopulmonary exercise testing

CPET was performed on an electronically braked cycle ergometer using a personalized ramp protocol set, to reach peak exercise in 10 ± 2 min (13) at T0 and applied unchanged at T2. CPET was performed and analyzed as standard (14). Specifically, in the absence of clinical events, tests were self-interrupted by patients when they reported the maximal effort. Patients wore a mask to measure ventilation (VE) and respiratory gases breath by breath. During the test, heart rate and a 12-lead ECG were continuously monitored, Hb O_2_ saturation was recorded by an oximeter, and blood pressure was monitored with a cuff sphygmomanometer at rest and every 2 min. PeakVO_2_ was calculated as the 30 s average of the highest VO_2_ recorded, while the VE/VCO_2_ slope was calculated based on the linear relationship between VE and VCO_2_, starting from 1 min after the initiation of loaded exercise until the end of the isocapnic buffering period. This value was also expressed as a percentage of the predicted value (15). Predicted peakVO_2_ was calculated using the Hansen and Wasserman equation as (height–age) × 20 for men and (height–age) × 14 for women (16). The anaerobic threshold (AT) was measured using a V-slope analysis of VO_2_ and VCO_2_ (17). The VO_2_/work relationship was measured through the entire exercise protocol. Other data are reported as the 20 s average.

The MECKI score, including six relevant prognostic parameters (Hb, LVEF, MDRD, Na, PeakVO_2_, and VE/VCO_2_ slope), was calculated as previously described (18).

### 2.3 Echocardiography

Transthoracic echocardiography (TTE) examinations were conducted using an Epiq CVx ultrasound machine (Philips Medical Systems, Andover, MA, USA) equipped with an X5-1 probe. A comprehensive standard 2D TTE analysis was performed, with left chamber volumes and LVEF measured from four-chamber and two-chamber views using the biplane Simpson’s method (19). All echocardiograms were conducted by highly trained operators. Pulmonary artery systolic pressure (PAP) was calculated by quantifying the peak velocity of tricuspid regurgitation and then adding the estimated pressure in the right atrium (20).

### 2.4 Bioelectrical impedance vector analysis

Bioimpedance measurements were conducted using an impedance plethysmograph (BIA 101 BIVA; AKERN SRL, Pisa, Italy) with a 250 µA RMS 50 kHz sinusoidal output signal. The device was calibrated using the standard control circuit with a known impedance [resistance = 383 ohms; reactance (Xc) = 45 ohms].

Measurements were taken with participants in a supine position, with their arms and legs by their sides. Values were recorded after a minimum rest of 5 min. Before measurement, the skin was cleaned with an alcohol solution and four contact electrodes (BIATRODES; AKERN SRL, Pisa, Italy) were placed on the dorsal surface of the right hand and foot as per the manufacturer's instructions.

KCCQ-12, CPET, spirometry, BIVA, and cardiac ultrasound were obtained and analyzed by medical personnel who were blinded to the study timeline, meaning they were unaware of whether the assessments were performed at T0, T1, or T2, while patients and referring physicians were unblinded.

The present research protocol complies with the World Medical Association’s Declaration of Helsinki and was approved by the Centro Cardiologico Monzino Ethical Committee (R 11637-22 CCM 1756). Each individual provided written informed consent to participate in the study. This study was registered on Clinicaltrials.gov (reference ID NCT05770167).

Study data were collected and managed using REDCap (Research Electronic Data Capture) electronic data capture tools hosted at Centro Cardiologico Monzino IRCCS (21, 22). REDCap is a secure, web-based software platform.

### 2.5 Statistical analysis

Continuous variables are described as mean ± standard deviation (SD) in case of normal distribution, and as median and interquartile range (IQR) in case of non-normal distribution. Categorical variables are expressed as numbers (percentages).

For continuous variables, differences between T0 and T6 were assessed with a paired *t*-test or a non-parametric test as appropriate. For categorical variables, McNemar’s test was used.

When variables were measured at all three protocol-specified time points (T0, T1, and T2), a statistical analysis was conducted using repeated measures tests for normally distributed variables or the Friedman test for non-normally distributed variables.

The correlation between the variables was evaluated using Pearson's correlation coefficient or Spearman’s non-parametric coefficient. A *p*-value <0.05 was considered statistically significant.

## 3 Results

In total, 75 patients have been enrolled between January 2022 and July 2023. Eight patients (10.7%) were excluded from the final analysis because they interrupted the drug or the study for personal reasons (specifically: two participants interrupted the study treatment for personal decision; six individuals continued the study treatment but did not perform the follow-up evaluation), while none of the enrolled patients stopped the treatment for clinical reasons or drug-related complains. All the remaining 67 HF patients (median age 66 years; age range 56–73 years) completed the evaluation at 6 months (T2), while 5 of 67 did not perform the safety evaluation at 2–4 weeks (T1). Table 1 reports the main parameters collected for the study population. At enrollment, all patients were on HF-optimized medical treatment, with 100% of patients were taking ACEi (*n* = 6, 9%), ARBs (*n* = 7, 10.4%), or Sacubitril/Valsartan (*n* = 54, 80.6%), 64 (95.5%) patients taking a *β*-blocker, 56 (83.6%) taking an MRA, and 35 (52.2%) taking a loop diuretic. With regard to comorbidities, at enrollment there were 7 (10.4%) patients with diabetes, 40 (59.7%) with dyslipidemia, 33 (49.3%) with hypertension, and 1 (1.5%) with moderate COPD. In total, 12 (18%) patients were current smokers while 26 (39%) were former smokers.

**Table 1:** Main variables at baseline (T0), 2–4 weeks (T1), and 6 months (T2).

Variable	*n*	T0	*n*	T1	*n*	T2	*p* T0 vs. T2	*p* Repeated meas.	Bonferroni *post-hoc* test		
									T2 vs. T0	T1 vs. T0	T1 vs. T2
Age (years)	67	66 [56–73]	67	66 [56–73]	66	66 [57–73]		0.604			
Weight (kg)	67	79.0 ± 14.3	61	79.1 ± 14.0	67	78.6 ± 14.2		0.016	–	0.020	–
Height (cm)	67	172 ± 8	67	172 ± 8	67	172 ± 8		1			
BMI (kg/m^2^)	67	26.5 ± 3.5	61	26.5 ± 3.4	67	26.4 ± 3.4		0.014	–	0.019	–
LVEF (mL)	67	34.6 ± 7.8			67	37.5 ± 9.2	<0.001				
EDV (mL)	67	186 [145–232]			67	177 [129–225]	<0.001				
ESV (mL)	67	113 [87–163]			67	110 [76–145]	<0.001				
PAPs (mmHg)	62	27.0 [23.7–29.0]			58	25.0 [23.0–28.0]	0.046				
SBP (mmHg)	67	118.6 ± 16.1				61	109.3 ± 13.4	<0.001			
DBP (mmHg)	67	72.5 ± 9.5			60	68.4 ± 7.9	0.003				
Heart rate (bpm)	63	63.4 ± 11.3			67	63.3 ± 12.0	0.796				
Vital capacity (L)	65	3.74 ± 0.95	60	3.84 ± 0.89	61	3.79 ± 0.90		0.672			
FEV1 (L)	67	2.71 ± 0.74	61	2.74 ± 0.74	62	2.69 ± 0.69		0.226			
FEV1 (%)	67	87.34 ± 17.04	61	88.18 ± 16.22	62	87.20 ± 15.19		0.358			
FVC (L)	67	3.38 ± 0.91	61	3.45 ± 0.93	62	3.43 ± 0.87		0.476			
FVC (%)	67	84.36 ± 16.11	61	84.58 ± 18.08	62	85.51 ± 14.82		0.772			
FEV1/FVC	67	0.80 ± 0.06	61	0.80 ± 0.07	62	0.79 ± 0.07		0.366			
Total body water (L)	67	44.7 [39.8–51]	60	44.9 [40.23–9.75]	67	44.9 [39.5–50]		0.145			
Extracellular water (L)	67	21.2 [18.2–23.1]	60	21.0 [18.82–2.95]	67	20.5 [18.4–22.9]		0.145			
Hydration index (%)	67	73.6 [73.2–73.8]	60	73.5 [73.3–73.8]	67	73.5 [73.3–73.8]		0.552			

BMI, body mass index; LVEF, left ventricular ejection fraction; EDV, left ventricle end-diastolic volume; ESV, left ventricle end-systolic volume; PAPs, systolic pulmonary artery pressure; SBP, systolic blood pressure; DBP, diastolic blood pressure; FEV1, forced expiratory volume in 1 s; FVC, forced vital capacity.

After 6 months of treatment with dapagliflozin, both systolic and diastolic blood pressure were lower than at T0, while heart rate was unchanged. Moreover, at the T2 visit, LVEF showed an 8% increase with a parallel reduction in LV volumes and a slight but significant decrease of PAPs (Table 1).

**Figure 2:**
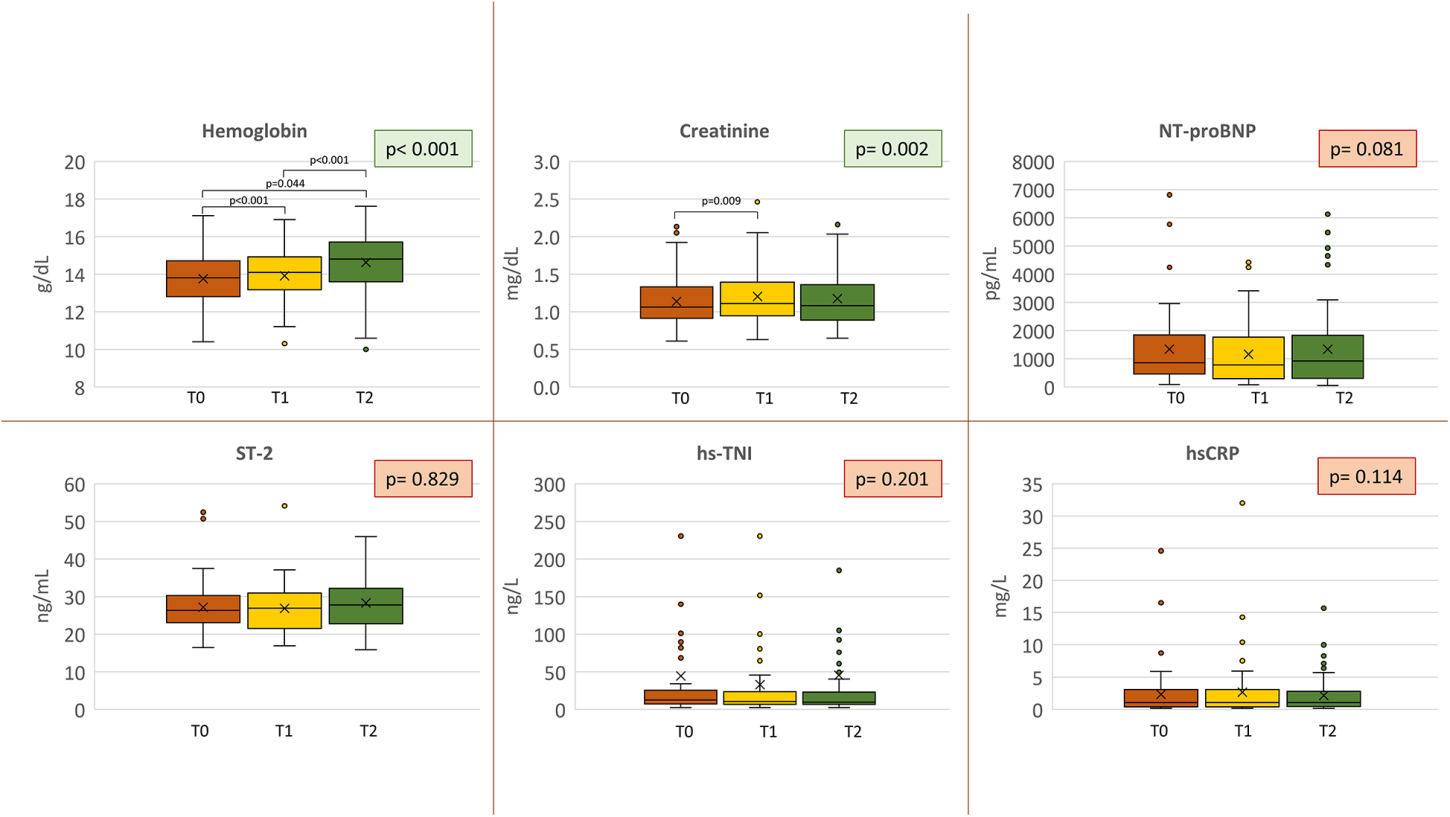
Biomarkers analysis. Biomarker variations at each study point. Orange = baseline; yellow = 2–4 weeks (T1); green = 6 months (T2). Nt-proBNP, N-terminal pro B-type natriuretic peptide; ST-2, suppression of tumorigenicity 2; hs-TNI, high-sensitivity troponin I; hsCRP, high-sensitivity C-reactive protein.

Medical treatment did not change during the course of the study, including the median dose of loop diuretics [25 mg/die (range 25–50) to 25 mg/die (range 25–25); *p* = 1.000]. None of the patients who were not on loop diuretics at baseline were prescribed them during the study, while one patient had loop diuretic therapy discontinued. Moreover, we detected a progressive increase of Hb (13.8 ± 1.5, 13.9 ± 1.5, and 14.6 ± 1.7 g/dL at T0, T1, and T2, respectively; *p* < 0.001) (Figure 2), and red cell distribution width (RDW) [13.7 (13.1–14.6), T1 13.8 (13.3–14.6), T2 13.8 (13.3–14.6); *p* < 0.019]. On the other hand, Na^+^ (140.3 ± 2.0, 140.1 ± 2.0, and 140.6 ± 2.2 at T0, T1, and T2, respectively; *p* = 0.192), NT-proBNP [from 852.0 pg/mL (455.3–1,845.3) at T0 to 916.5 pg/mL (301.7–1,831.0) at T2; *p* = 0.081], interleukin ST-2 [from 26.35 ng/mL (23.08–30.23) to 27.80 ng/mL (22.80–32.10); *p* = 0.829], hsCRP [from 1.07 mg/L (0.40–3.07) to 1.04 mg/L (0.46–2.82); *p* = 0.114], and hs-TNI [from 12.22 ng/L (7.31–25.53) to 9.49 ng/L (6.74–22.88); *p* = 0.201] did not significantly change during the study (Figure 2). We observed a short-term worsening of creatinine (T1) with a complete recovery of values at T2 (*p* = 0.009 T0 vs. T1) (Figure 2). The same temporal trend was confirmed if renal function was analyzed as MDRD (73.0 ± 22.8, 68.8 ± 21.3, and 70.2 ± 20.9 at T0, T1, and T2, respectively; *p* < 0.01).

**Figure 3:**
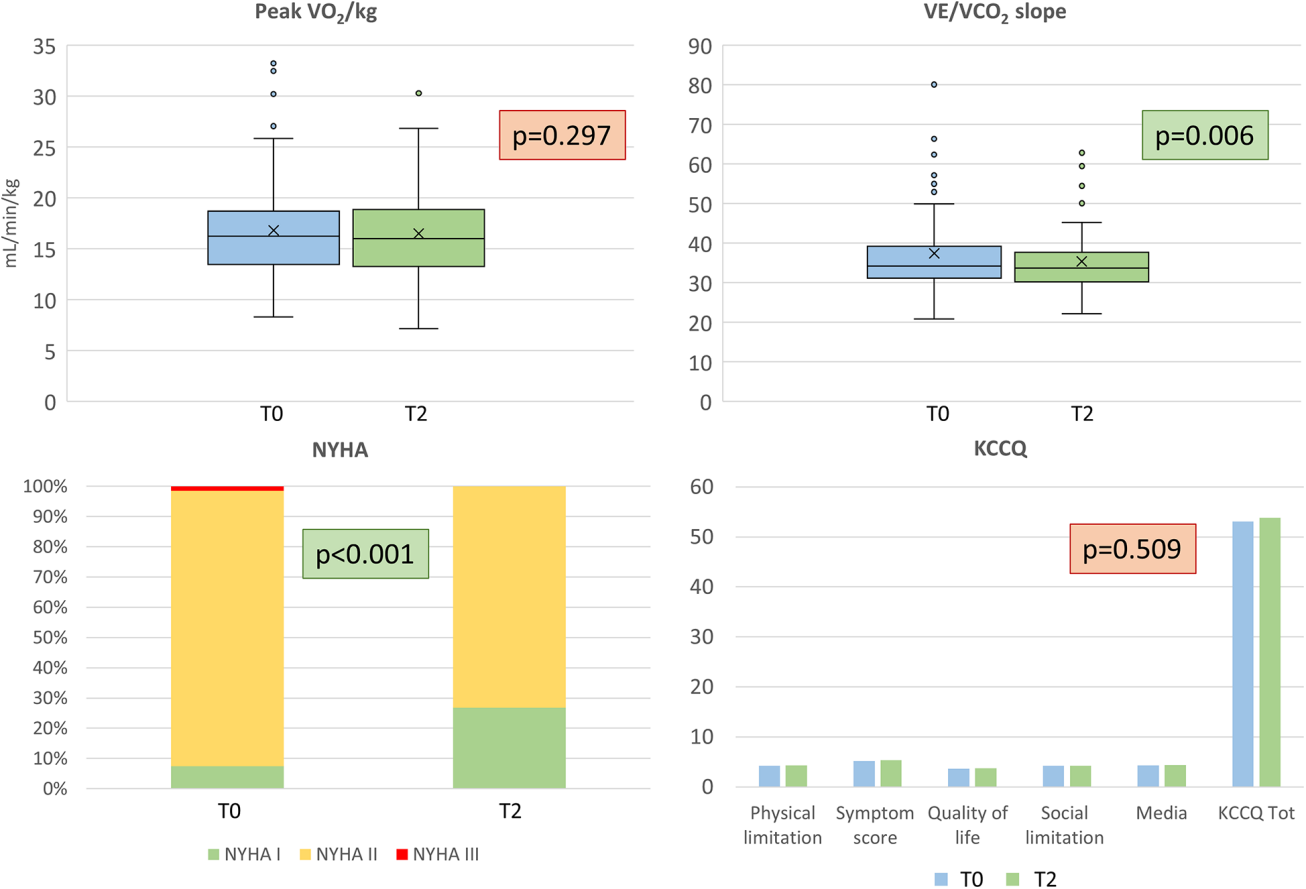
Functional and quality of life evaluation. Top: peak oxygen uptake (VO_2_/kg) on the left, and ventilatory efficiency (VE/VCO_2_ slope), on the right. Bottom: New York Heart Association (NYHA) class and Kansas City Cardiomyopathy Questionnaire results are reported on the left and right respectively. T0 = baseline, T2 = 6 months.

Regarding CPET evaluation, no significant changes in terms of peakVO_2_ and other VO_2_-derived CPET parameters were detected at 6 months, while the VE/VCO_2_ slope reduced both as absolute value and as percent of predicted (Table 2, Figure 3). Notably, two patients did not repeat the cardiopulmonary assessment at 6 months due to limitations not related to HF; therefore, they were excluded from the present analysis.

**Table 2:** Functional and QoL evaluation of study population at baseline (T0) and after 6 months (T2).

Variable	*n*	T0	*n*	T2	*p*
NYHA I	67	5 (7.5%)	67	18 (26.9%)	<0.001
NYHA II-III	67	62 (92.5%)	67	49 (73.1%)	
VO_2_-AT (mL)	60	885.2 ± 268.9	61	874.9 ± 265.6	0.386
VO_2_/kg-AT (mL/kg)	60	11.3 ± 3.1	61	11.1 ± 2.6	0.345
Heart rate-AT (bpm)	60	87.33 ± 16.26	61	86.15 ± 15.41	0.529
PeakVO_2_ (mL/min)	65	1,212 [996–1,593]	65	1,246 [993–1,583]	0.190
PeakVO_2_ (mL/min/kg)	65	16.21 [13.43–18.67]	65	15.98 [13.26–18.85]	0.297
PeakVO_2_ (% pred)	65	64.3 ± 17.1	65	63.1 ± 16.5	0.375
Peak heart rate (bpm)	65	114 ± 25	65	115 ± 25	0.586
Peak workload (watt)	65	112.0 [84.5–128.5]	65	109.0 [78–141.5]	0.297
Peak pulse (mL/beat)	65	11.71 ± 3.25	65	11.43 ± 3.35	0.251
Peak Systolic blood pressure (mmHg)	65	150 [130–170]	65	140 [127–160]	0.264
VE/VCO_2_ slope	65	34.2 [31.1–39.2]	65	33.7 [30.2–37.6]	0.006
VE/VCO_2_ slope (% pred)	65	130 [117–147]	65	126 [115–140]	0.003
VO2/work slope (mL/min/watt)	65	9.21 [7.86–9.78]	65	9.08 [8.30–9.74]	0.799
Peak respiratory rate (L/min)	65	35.09 ± 7.25	65	34.54 ± 6.88	0.422
Peak ventilation (L/min)	65	62.37 ± 19.17	64	60.76 ± 20.31	0.527
Rest PetCO_2_ (mmHg)	65	28.0 ± 3.3	65	27.9 ± 3.8	0.750
PetCO_2_-AT (mmHg)	60	34.8 ± 4.7	61	35.0 ± 4.7	0.628
PetCO_2_-RCP (mmHg)	50	33.9 ± 5.0	44	34.2 ± 5.4	0.953
Peak PetCO_2_ (mmHg)	65	30.3 ± 4.8	65	30.7 ± 4.9	0.225
Peak respiratory exchange ratio	65	1.12 ± 0.12	65	1.13 ± 0.14	0.568
KCCQ-Physical limitation	64	4.20 ± 0.9	63	4.29 ± 0.85	0.404
KCCQ-Symptoms	64	5.19 ± 0.96	63	5.30 ± 0.91	0.438
KCCQ-QoL	64	3.66 ± 1.08	63	3.70 ± 1.12	0.847
KCCQ-Social limitation	64	4.17 ± 1.09	63	4.22 ± 1.11	0.789
KCCQ-Mean	64	4.31 ± 0.88	63	4.38 ± 0.87	0.529
KCCQ-Total	64	53.1 ± 10.4	63	53.8 ± 10.3	0.509

NYHA, New York Heart Association; VO_2_, oxygen intake; %pred, percentage of predicted value; AT, anaerobic threshold; VE/VCO_2_, minute ventilation/carbon dioxide production relationship; PetCO_2_, end tidal pressure of CO_2_; RCP, respiratory compensation point; KCCQ, Kansas City Cardiomyopathy Questionnaire; QoL, quality of life.

KCCQ did not reveal an improvement in the subjective perception of QoL. No significant differences with respect to the T0 evaluation were found in either the total score or in the analyzed domains (Table 2, Figure 3). VE/VCO_2_ slope and Hb improvements were not correlated (*R*^2^ for ΔVE/VCO_2_ vs. ΔHb 0.029). NYHA class improved in 13 patients (from NYHA II or III at baseline to NYHA I at T2) (*p* <0.001). Only one patient worsened from NYHA I to NYHA II. Regarding BIVA, we did not find any significant difference (Table 1). We observed a significant improvement in median MECKI score (18) from 3.3% (range 1.9–8.0) to 2.8% (range 1.2–5.7), suggestive of a positive impact on prognosis at 2 years (*p* <0.001).

## 4 Discussion

The main finding of this study is that in our cohort of HFrEF patients, dapagliflozin had no impact on exercise capacity, as assessed by peakVO_2_ and workload. Similarly, other relevant CPET parameters, including VO_2_ at AT, VO_2_/work, peakO_2_-pulse, and peak heart and respiratory rates, remained unchanged, with patients achieving maximal or near maximal effort (Table 2). However, dapagliflozin improved exercise ventilatory efficiency, as shown by a modest but significant VE/VCO_2_ slope reduction (34.2 vs. 33.7; *p* < 0.001).

The neutral effect of the drug on peakVO_2_ is surprising, given previously reported data. The DAPA-VO_2_ study (23) documented a significant improvement in peakVO_2_ after 1 and 3 months of treatment (+Δ 1.09 mL/kg/min and +Δ 1.06 mL/kg/min, respectively). Compared to that trial, our cohort had less advanced HF, as indicated by the relatively higher baseline peakVO_2_ (16.2 mL/kg/min vs. 13.4 mL/kg/min), LVEF (34.6% vs. 33.7%), and lower NT-proBNP levels (774 vs. 1,085 pg/ml). Therefore, a smaller effect on peakVO_2_ in our population may have been expected. The lack of peakVO_2_ improvement with dapagliflozin remains unexpected, especially considering the increases in LVEF and Hb. O_2_ delivery is directly linked to cardiac output (CO) and Hb levels. In this study, the former was possibly unchanged, being the improvement of LVEF obtained in parallel with the reduction in LV volumes, while Hb was undoubtedly and significantly increased by dapagliflozin (from 13.8 to 14.6 g/dL; *p* < 0.001), consistent with previous studies (24, 25). We hypothesize two possible explanations for this. First, the reduction in LV volumes might have led to a relevant decrease in peak CO regardless of the improvement in LVEF, though this seems unlikely. Alternatively, dapagliflozin may have influenced exercise-induced blood flow redistribution. This phenomenon, which we recently reported, is rarely considered in clinical practice but may explain the blunted changes in peakVO_2_, despite significant hemodynamic improvement in HF patients receiving effective treatments (26). Indeed, as the severity of HF increases, blood flow distribution during exercise is, as a percentage of total blood flow, progressively directed toward the working muscles, leading to differences in increased arteriovenous O_2_ content (Δa-vCO_2_). However, as HF improves and CO increases, the percentage of blood flow to the muscle decreases, leading to a reduction in Δa-vCO_2_ and affecting peakVO_2_ measurements. This phenomenon may explain the discrepancy observed between peakVO_2_ and LVEF/Hb changes. Therefore, dapagliflozin’s improvement on exercise hemodynamics cannot be ruled out by the unchanged peakVO_2_ we observed.

The reduction of the VE/VCO_2_ slope, indicating improved VE efficiency during exercise, is another interesting observation. Indeed, the VE/VCO_2_ slope is an important parameter directly related to HF prognosis in HFrEF as well as in other cardiomyopathies (27, 28) with a prognostic significance comparable to peakVO_2_ (29). Therefore, it is included in heart transplant screening guidelines (30) and in HF prognostic scores involving exercise evaluation, such as the MECKI score (18). The VE/VCO_2_ slope depends on chemoreflex-mediated VE regulation and VE/perfusion mismatch in the lungs. While there are no available data on the direct effects of SGLT-i on chemo- or metaboreceptor activity, the absence of changes in PetCO_2_ at rest, during exercise, and specifically at AT, respiratory compensation point, and peak exercise, suggests a change in the effects of reflex on ventilation during exercise. An improvement in VE/perfusion mismatch at the lung level seems likely, as pulmonary pressure was significantly reduced in the cardiac ultrasound evaluation. This postulated hemodynamic improvement may be due to the well-known dapagliflozin-related diuretic effect (31), which helps reduce pulmonary pressures and interstitial edema. However, our data do not support a relevant diuretic effect, as patients' weight loss was negligible (−0.4 kg) and NT-proBNP and BIVA (i.e., overall hydration index and body water) remained unchanged (Table 1, Figure 2). Notably, BIVA does not assess thoracic fluids. Therefore, although the overall hemodynamic effect of dapagliflozin at rest is limited, an improvement during exercise may still be possible.

In addition to exercise, we demonstrated a favorable LV reverse remodeling with a statistically significant (though small) reduction in both EDV and ESV, along with an improvement in LVEF (Table 1). These changes are in line with previous data (9, 32, 33) and confirm the positive effects of SGLT2-i on LV geometry and HF progression. Prevention and reversal of adverse cardiac remodeling is one of the mechanisms through which SGLT2-i may exert cardiovascular benefits, involving molecular, cellular, and interstitial changes related to increased apoptosis and necrosis, decreased autophagy, impairments of myocardial oxygen supply and demand, and altered energy metabolism (32). However, in the present study, these positive changes in cardiac volumes and function were not accompanied by significant improvements in cardiac biomarkers. In fact, NT-proBNP, hs-TNI, and ST-2 levels did not change.

The results of the present trial should be considered in the context of existing data. Most of our patients were non-diabetic and NYHA class II at baseline. The KCCQ demonstrated a mild degree of QoL impairment; similarly, peakVO_2_ and NT-proBNP values indicate a non-severe spectrum of HF. Importantly, our population had a higher degree of HF therapeutic optimization with disease-modifying therapy compared to most studies. For example, in the DAPA-HF study (1), only 10% of the patients were taking sacubitril/valsartan, compared to 81% of our population. This, together with the other standard HF therapies, contributed to a baseline mild impairment in peakVO_2_ (16.21 mL/kg/min). Moreover, the same trial observed a significant reduction in NT-proBNP (−196 ± 2,387 pg/mL), but from slightly higher baseline values. In fact, other studies conducted in specific HF phenotypes (e.g., amyloidosis) have confirmed a positive effect of dapagliflozin on NT-proBNP (34). In other words, the drug’s impact on biomarkers related to LV stretch/overload seems to depend on the baseline value and/or on the length of the follow-up. Therefore, it is difficult to expect further clinical benefits in this parameter in a population with stable non-severe HF.

**Figure 4:**
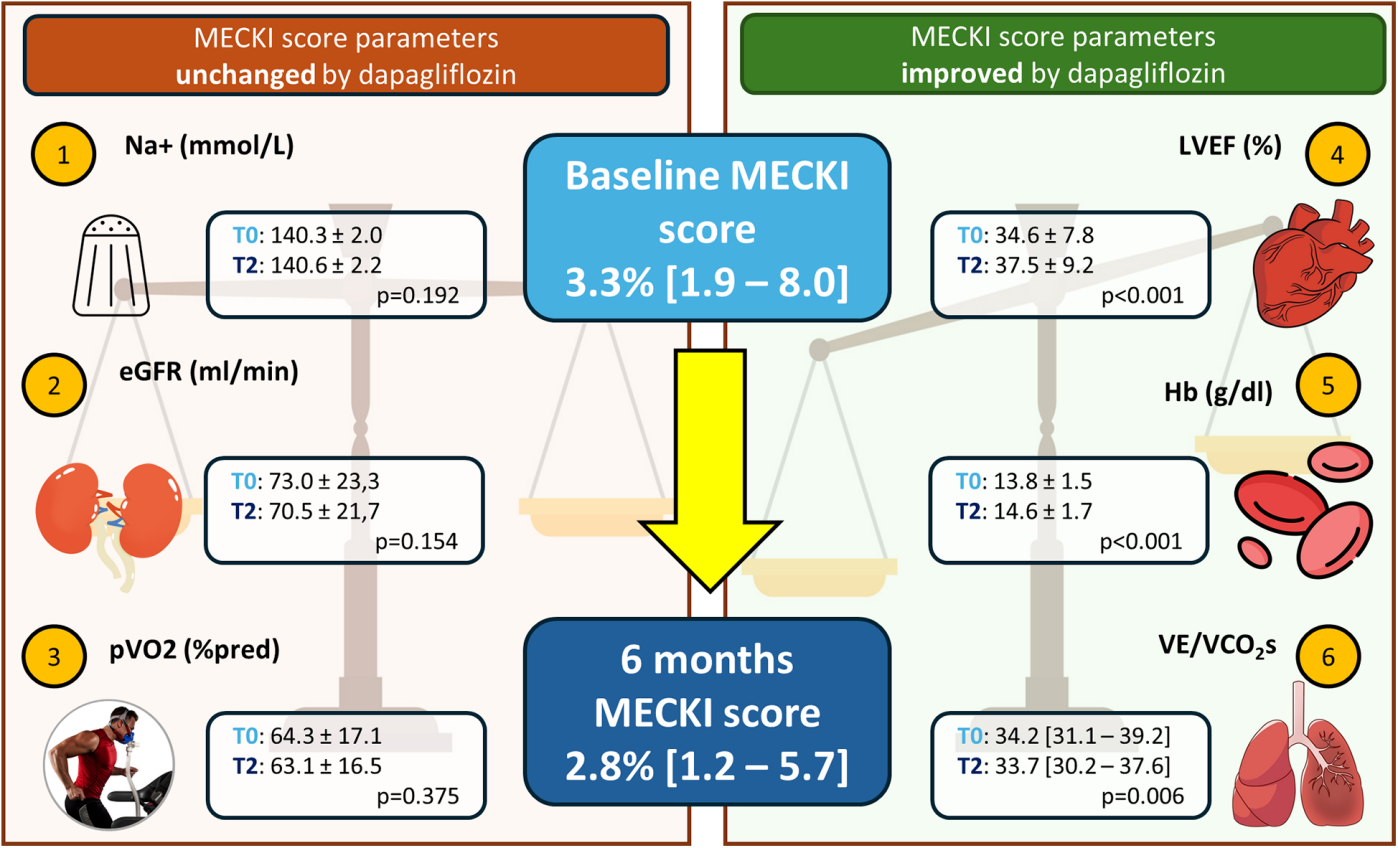
Effect of dapagliflozin on prognosis estimation by MECKI score. Schematic representation of the six variables constituting the MECKI score, which assesses prognosis. Left: variables that remained unchanged in our study; right: variables that significantly improved after 6 months with dapagliflozin. Na+, sodium; eGFR, estimated glomerular filtration rate calculated by modification of diet in renal disease (MDRD) formula; MECKI, Metabolic Exercise test data combined with Cardiac and Kidney Index; pVO_2_ (%pred), peak oxygen uptake expressed as_%_ of predicted; LVEF, left ventricular ejection fraction; Hb, hemoglobin; VE/VCO_2_s, ventilatory efficiency.

In a population with moderate HF and uptitrated treatment, dapagliflozin does not seem to have additional effects on top of sacubitril/valsartan in terms of peakVO_2_ and cardiac biomarkers, which are considered pivotal for assessing HF treatment efficacy. However, these mechanistic findings, which may initially seem disappointing, should not limit the use of SGLT2-i. Even in our well-treated, stable, low-severity population, we observed an additional favorable effect on the prognostic balance, as shown by a significant improvement in the MECKI score (Figure 4). This confirms that the evaluation of HF patients should not be limited to a single variable, even if it is prognostically important (e.g., peakVO_2_), but requires a holistic evaluation. Furthermore, dapagliflozin demonstrated excellent tolerability without significant side effects across the entire population. Finally, it is also important to remember that HF is a progressive and debilitating disease characterized by the worsening of parameters over time (including peakVO_2_ and biomarkers). Therefore, even “freezing” the situation, as we did in the present study, can be considered a success.

### 4.1 Limitations

This study has some limitations. First, due to ethical reasons, it is randomized, meaning a direct comparison of interventions is not possible. Second, the monocentric nature of the study with a small sample size limits the automatic generalization of the results to other populations. Third, most of the patients were in NYHA class II, with relatively stable, non-advanced heart failure. Therefore, the effects of the drug in more severe HF populations deserve to be studied in dedicated trials. Fourth, the study was designed to detect changes during the first few months of treatment in patients with reduced LVEF. Further studies are needed to analyze the long-term effects and outcomes in other HF groups (with midrange or preserved LVEF). Finally, we only studied patients treated with dapagliflozin, not other SGLT2-i. Therefore, we do not know whether similar results can be obtained with different SGLT2-i.

In conclusion, our trial highlighted a beneficial impact of dapagliflozin on key HF parameters, such as VE/VCO_2_ slope, Hb, LV volumes, and ejection fraction, despite a neutral effect on peakVO_2_ and cardiac biomarkers. These findings help to understand the type of benefits to expect from this pillar of HF therapy, even in a well-treated population of clinically stable patients with moderate HFrEF.

## Data Availability

The raw data supporting the conclusions of this article will be made available by the authors, without undue reservation.
